# OsMKKK70 Negatively Regulates Cold Tolerance at Booting Stage in Rice

**DOI:** 10.3390/ijms232214472

**Published:** 2022-11-21

**Authors:** Enyang Mei, Jiaqi Tang, Mingliang He, Zhiqi Liu, Xiaojie Tian, Qingyun Bu

**Affiliations:** 1 Key Laboratory of Soybean Molecular Design Breeding, Northeast Institute of Geography and Agroecology, Chinese Academy of Sciences, Harbin 150081, China; 2 University of Chinese Academy of Sciences, Beijing 100049, China; 3 Key Laboratory of Saline-Alkali Vegetation Ecology Restoration (Northeast Forestry University), Ministry of Education, Harbin 150040, China; 4 College of Life Science, Northeast Forestry University, Harbin 150040, China; 5 The Innovative Academy of Seed Design, Chinese Academy of Sciences, Beijing 100101, China

**Keywords:** cold tolerance at booting stage, gibberellin, OsMKKK70, rice

## Abstract

Cold stress at the booting stage leads to a lower seed setting rate and seriously threatens the production of rice (*Oryza sativa* L.), which has become a major yield-limiting factor in higher-altitude and -latitude regions. Because cold tolerance at the booting stage (CTB) is a complex trait and is controlled by multiple loci, only a few genes have been reported so far. In this study, a function of OsMKKK70 (Mitogen Activated Protein Kinase Kinase Kinase 70) in response to CTB was characterized. *OsMKKK70* expression was rapidly induced by cold stress at the booting stage. *OsMKKK70* overexpression (*OsMKKK70-OE*) plants were more sensitive to cold stress at the booting stage with a lower seed setting and pollen fertility, but there was no significant difference between the *osmkkk70* mutant and WT. Considering the effect of functional redundancy, we further tested the CTB response of *osmkkk62/70* and *osmkkk55/62/70*, the double and triple mutants of *OsMKKK70* with its closest homologs *OsMKKK62* and *OsMKKK55*, and found that *osmkkk62/70* and *osmkkk55/62/70* displayed significantly increased CTB with a higher seed setting and pollen fertility, indicating that OsMKKK70 negatively regulates rice CTB. Moreover, under the low-temperature (LT) condition, the *osmkkk62/70* mutant had slightly higher Gibberellin (GA) contents, increased expression of GA biosynthesis genes, and lower protein level of OsSLR1 in anthers than those in WT. By contrast, *OsMKKK70-OE* anther had a lower GA biosynthesis than that of WT. Together, these findings suggest that OsMKKK70 negatively regulates rice CTB by fine-tuning GA levels in anthers.

## 1. Introduction

Rice is an important cereal crop and widely cultivated around the world. Cold tolerance at the booting stage (CTB) is a major limiting factor for rice production and geographic distribution [1,2,3,4]. Cold stress at the booting stage severely injures microspore development and pollen fertility, which seriously threaten the yield and quality of rice [3]. Cold stress at the booting stage is more severe and frequent in high-latitude and high-altitude regions. In China, the annual loss of rice production due to low temperature is as high as 3–5 million tons [4]. Therefore, it is an urgent task to improve cold tolerance at the booting stage for rice breeding.

Cold tolerance is a complex quantitative trait and is controlled by multiple genes [5]. Cold stress at the booting stage is challenging in terms of phenotypic evaluation, which limits the identification of cold-related genes or the dissection of the underlying molecular mechanisms. Therefore, although some multiple QTLs controlling CTB have been mapped, only a few genes, including *CTB1*, *CTB2*, *CTB4a*, *OsbZIP73*, *LTT1*, *OsWRKY53*, *OsLEA9*, and *OsMAPK3*, have been cloned in the past decades [2,3,5,6,7,8,9,10,11]. *CTB2*, encoding a UDP-glucose sterol glucosyltransferase, positively regulates cold tolerance via mediating sterol metabolism to maintain cell membrane permeability and protect the pollen grain and pollen wall from cold damage [12,13]. Natural variation in the *CTB4a* promoter in *Japonica* rice enhances *CTB4a* expression, and up-regulation of *CTB4a* correlates with increased ATP synthase activity, ATP content, enhanced seed setting, and improved yield under cold stress conditions [5]. *OsbZIP73* controls cold tolerance at both the seedling and booting stages by regulating abscisic acid (ABA) and reactive oxygen species (ROS) levels in plant tissues [2,14]. *LTT1* negatively regulates cold tolerance at the booting stage by maintaining ROS homeostasis [3]. *OsWRKY53* negatively regulates rice CTB by fine-tuning GA levels in anther [9]. *OsMAPK3* regulates cold tolerance at both the seedling and booting stages by the trehalose biosynthetic pathway [7,11]. *CTB1* and *OsLEA9*, respectively, encode an F-box protein and late embryogenesis abundant protein that controls CTB, but the underlying mechanism remains elusive [7,8,15]. Functional dissection of these genes will largely contribute to the improvement of CTB in rice and expand the geographic distribution of rice cultivation.

Phytohormone gibberellic acid (GA) is an essential determinant of growth and development in rice [4,16,17,18]. During the past decades, many mutants defective in GA signaling or biosynthesis have been identified in rice. Most of the GA-related mutants display poorly developed anthers and a lower seed setting rate, suggesting that GA plays critical roles in regulating rice reproductive development [16,19]. It has been reported that GA was associated with cold tolerance at the booting stage in rice, low-temperature treatment markedly decreased GA contents in anthers, and that the application of exogenous GA can successfully rescue cold-injured pollen sterility [9,20].

Mitogen-activated protein kinase (MAPK) cascades are important signaling modules that play vital roles in diverse processes of plant growth and development [21]. A typical MAPK cascade is composed of three kinds of protein kinases: MAPK (MPK), MAPK kinase (MKK), and MKK kinase (MKKK). In response to a stimulus, the MAPK cascade module transfers signals through a relay of phosphorylation events. In *Arabidopsis*, the involvement of MAPK signaling has been found to regulate the cold stress response via the ICE1 pathway, in which MPK3 and MPK6 regulate the protein stability of ICE1 via phosphorylating ICE1, and then negatively regulate the cold tolerance [22,23]. YDA, a MAPKKK, and two calcium/calmodulin-regulated receptor-like kinases CRLK1 and CRLK2 were also involved in the cold response via negatively regulating the cold activation of MPK3/MPK6 in *Arabidopsis* [23]. Moreover, the MEKK1-MKK2-MPK4/MPK6 cascade positively regulates the cold response and freezing tolerance in *Arabidopsis* as well [24]. In rice, OsMAPK3 was shown to positively regulate cold tolerance at the seedling stage and reproductive stage [7,11]. OsMAPK3 phosphorylates and stabilizes OsICE1/OsbHLH002, and then activates OsTPP1 and enhances rice chilling tolerance [11]. Rice contains approximately 75 OsMKKKs family members that have diverse functions in different signaling process [25,26,27], whereas the involvement of OsMKKKs in regulating cold tolerance has not been reported.

We previously demonstrated that OsMKKK70 regulates rice grain size and leaf angle through the OsMKK4-OsMAPK6-OsWRKY53 cascade signaling pathway [6]. Recently, we also reported that OsWRKY53 negatively regulates CTB via tuning the GA level in anthers [9]. These results have motivated us to investigate whether OsMKKK70 is involved in rice CTB. In this study, we discovered that OsMKKK70 is a novel negative regulator of CTB by modulating the GA content.

## 2. Results

### 2.1. OsMKKK70 Might Be a Novel Regulator of Cold Tolerance at Booting Stage

**Figure 1:**
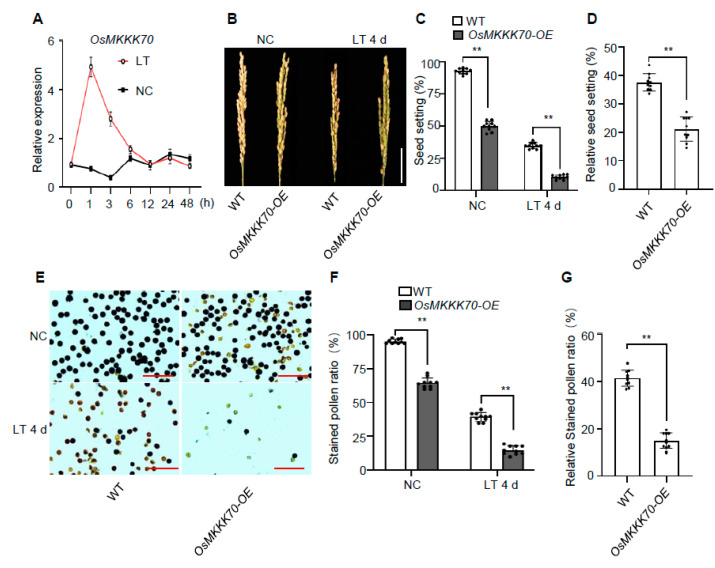
OsMKKK70 is involved in cold tolerance at the booting stage in rice. (**A**) Time course of *OsMKKK70* relative expression in panicles of WT exposed to a 15 °C cold treatment during the booting stage. The expression level on 0 h was set to 1. Data are shown as means ± SE (n = 3). (**B**–**D**) Representative images of panicles (**B**), seed setting rate (**C**), and relative seed setting rate (**D**) of WT and *OsMKKK70-OE* plants grown under NC, and after LT treatment for 4 days. The scale bar in (**B**) is 5 cm. Data are shown as means ± SE (n = 10). (**E**) Representative images of stained pollen from WT and *OsMKKK70-OE* plants grown under NC, and after LT treatment for 4 days. The scale bars are 50 μm. (**F**) Quantification of stained pollen ratio in (**E**). Data are shown as means ± SE (n = 10). (**G**) Quantification of relative stained pollen ratio in (**E**). Data are shown as means ± SE (n = 10). Each dot represents the result from one biological replicate, and error bars indicate means ± SE. *p* values were calculated by Student’s *t*-test; ** *p* < 0.01.

Cold stress at the booting stage is an important abiotic stress that severely affects the production and geographic distribution of rice [1,2,3,4]. To explore the cold-tolerance-related genes at the booting stage and the underlying regulatory pathways, we performed transcriptome analysis using the young rice panicles exposed to the low temperature (LT) of 15 °C at the booting stage. We identified approximately 1000 differentially expressed genes, and *OsMKKK70* was one of LT-induced expressed genes. To confirm this result, we re-examined the expression of *OsMKKK70* treated with LT at the booting stage, and found that *OsMKKK70* was rapidly induced, peaked at 1 h of LT treatment, and showed a gradual descent thereafter (Figure 1A). To study the biological function of OsMKKK70, the *OsMKKK70* overexpression plants (*OsMKKK70-OE*) were generated [6]. Interestingly, *OsMKKK70-OE* plants exhibited obviously shriveled and pale anther morphology and lower seed setting rate at the normal condition (NC), which is similar to wild type (WT) subjected to LT injury, implying that OsMKKK70 might be involved in cold tolerance at the booting stage (CTB) [6]. We, therefore, selected *OsMKKK70* for further characterization in this study.

First, we carefully examined the seed setting rate of *OsMKKK70-OE* and showed that it was significantly lower than those of the WT in both NC and LT conditions (Figure 1B,C). After LT for 4 d at 15 °C, the relative seed setting rate of the *OsMKKK70-OE* plant was around 21.1%, while that of WT was around 38% (Figure 1D). This result indicated that *OsMKKK70-OE* is hypersensitive to cold stress at the booting stage. Cold stress at the booting stage usually affects anther development and pollen fertility in rice, which results in a decreased seed setting rate [15,20]. Therefore, we also observed the pollen fertility and anther morphology of *OsMKKK70-OE*. It was shown that, under NC, the pollen fertility of *OsMKKK70-OE* was lower than that of WT (Figure 1E,F). Upon LT treatment, the pollen fertility of the *OsMKKK70-OE* was even much lower than that of WT (Figure 1E–G), which was consistent with the lower seed setting rate of the *OsMKKK70-OE* plant (Figure 1B–D). In addition, under NC, the anther of *OsMKKK70-OE* was shriveled and pale, compared with the normal anther of WT (Figure S1A). Upon LT treatment, the degree of shrunken and pale of *OsMKKK70-OE* anther was further enhanced (Figure S1A). Collectively, these results indicated that overexpressing *OsMKKK70* leads to more sensitivity to cold stress at the booting stage, suggesting that OsMKKK70 is involved in rice CTB.

### 2.2. OsMKKK70 Negatively Regulates Rice CTB

To confirm that OsMKKK70 is involved in CTB, we generated *osmkkk70* mutants via CRISPR/Cas9-mediated genome editing [6]. The *osmkkk70* mutant showed a comparable seed setting rate to WT under NC and LT treatment (Figure S2A–C). Our previous work demonstrated that *OsMKKK70* has two homologs (*OsMKKK62* and *OsMKKK55*) with high sequence similarity and these three OsMKKK genes function redundantly [6]. Therefore, we also examined the cold tolerance of *osmkkk55* and *osmkkk62* single mutants, and found that *osmkkk55* and *osmkkk62* showed comparable cold tolerance to WT (Figure S2D–I).

**Figure 2:**
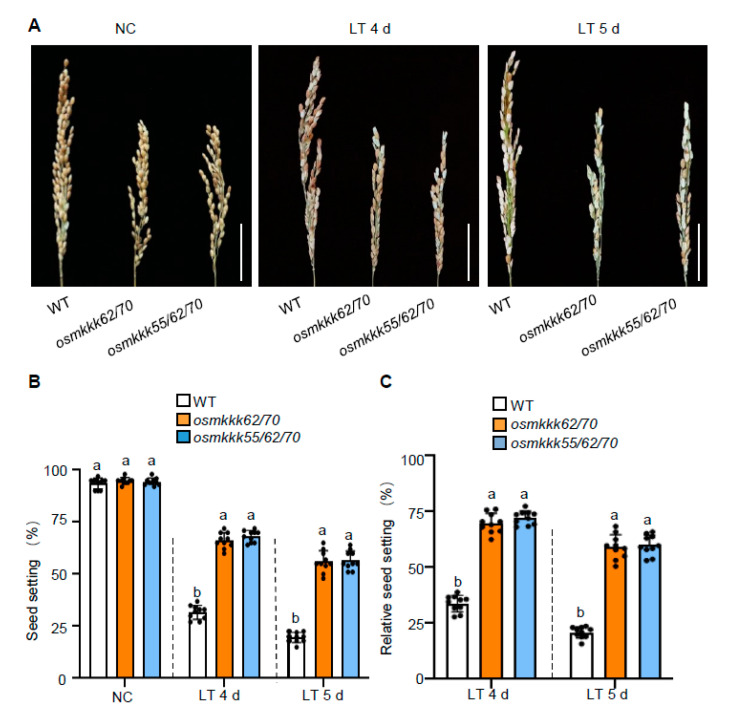
*osmkkk62/70* and *osmkkk55/62/70* mutants show increased cold tolerance at booting stage. (**A**) Representative images of panicles of WT, *osmkkk62/70*, and *osmkkk55/62/70* mutants grown under NC, and after LT treatment for 4 or 5 days. The scale bars are 5 cm. (**B**,**C**) Seed setting rate (**B**) and relative seed setting rate (**C**) of WT, *osmkkk62/70*, and *osmkkk55/62/70* mutants grown under NC, and after LT treatment for 4 or 5 days. Data are shown as means ± SE (n = 10). Dotted lines were drawn to separate the NC and LT, and the comparison is among different genotypes in the same condition. Each dot represents the result from one biological replicate; error bars indicate means ± SE. Statistically significant differences are indicated by different lowercase letters (*p* < 0.05, one-way ANOVA with Tukey’s significant difference test).

To further explore the biological function of OsMKKK70 in response to CTB, *osmkkk62/70* double and *osmkkk55/62/70* triple mutants were used for the cold stress assay [6]. *osmkkk62/70* and *osmkkk55/62/70* mutants had comparable seed setting rates to WT under NC (Figure 2A,B). However, under LT conditions, *osmkkk62/70* and *osmkkk55/62/70* mutants exhibited much higher seed setting rates and relative seed setting rates than those of the WT (Figure 2A–C). Indeed, the relative seed setting rate of the *osmkkk62/70* was 69.8% and 59.2% after LT for 4 or 5 d at 15 °C, respectively; the *osmkkk55/62/70* mutant was 72.2% (after LT for 4 d) and 60.1% (after LT for 5 d); while that of WT only reached 33.9% (after LT for 4 d) and 20.9% (after LT 5 d) (Figure 2C). To confirm that *osmkkk62/70* and *osmkkk55/62/70* mutants increased the CTB, we performed two more independent replications in two consecutive years from 2019 to 2020. It was shown that *osmkkk62/70* and *osmkkk55/62/70* mutants reproducibly displayed a higher seed setting rate than WT in LT treatment (Figure S3A–D). Combined with the *OsMKKK70-OE* being hypersensitive to LT and *osmkkk62/70* and *osmkkk55/62/70* mutants having increased CTB, these results suggest that OsMKKK70 plays the major role in negatively regulating rice CTB, and OsMKKK55 and OsMKKK62 might have minor and redundant functions in this process.

### 2.3. OsMKKK70 and OsMKKK62 Affect Pollen Fertility and Anther Development

**Figure 3:**
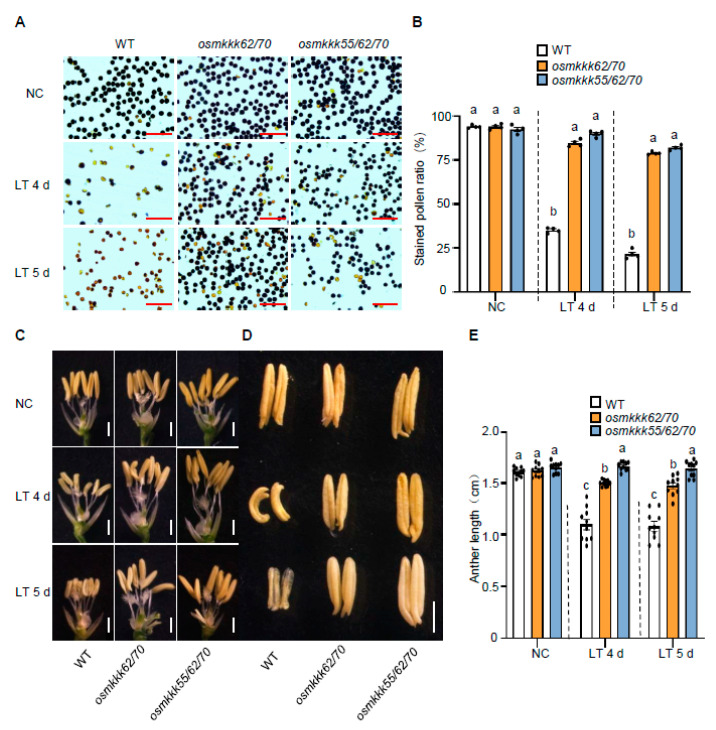
*osmkkk62/70* and *osmkkk55/62/70* show higher pollen fertility and healthy anthers under low-temperature treatment. (**A**) Representative images of stained pollen from WT, *osmkkk62/70*, and *osmkkk55/62/70* plants grown under NC, and after LT treatment for 4 or 5 days. The scale bars are 50 μm. (**B**) Quantification of stained pollen ratio in (**A**). Data are shown as means ± SE (n = 10). (**C**,**D**) Gross morphology of anthers from WT, *osmkkk62/70*, and *osmkkk55/62/70* plants grown under NC and exposed to 4 or 5 d of LT treatments. The scale bar in (**C**,**D**) is 1 cm and 5 mm, respectively. (**E**) Quantification of anther length of WT, *osmkkk62/70*, and *osmkkk55/62/70* plants grown under NC and exposed to 4 or 5 d of LT treatments. Values are means ± SE (n = 10). Dotted lines were drawn to separate the NC and LT, and the comparison is among different genotypes in the same condition. Each dot represents the result from one biological replicate; error bars indicate means ± SE. Statistically significant differences are indicated by different lowercase letters (*p* < 0.05, one-way ANOVA with Tukey’s significant difference test).

It has been proposed that anther length and anther morphology are positively correlated with CTB and can be used as criteria for evaluating CTB in rice [9,13]. To study how OsMKKK70 negatively regulates rice CTB, we examined the pollen fertility and the anther morphology of *osmkkk62/70* and *osmkkk55/62/70*. It was shown that, under NC, the pollen fertility of *osmkkk62/70* and *osmkkk55/62/70* mutants was comparable to WT plants (Figure 3A,B). By contrast, under LT treatment, the fertile pollen ratio of *osmkkk62/70* and *osmkkk55/62/70* mutants was much higher than that of WT plants (Figure 3A,B), which was consistent with the higher seed setting rate of *osmkkk62/70* and *osmkkk55/62/70* mutants under LT treatment (Figure 2A–C and Figure S3A–D). For anther morphology, after exposure to LT treatment for 4 or 5 d, WT plants anthers had a shorter, distorted, and deformed morphology and the color was light yellow (Figure 3C–E). In contrast, the anthers of *osmkkk62/70* and *osmkkk55/62/70* mutants remained bright yellow and longer after 4 and 5 d of 15 °C treatment (Figure 3C–E). Together, these results indicated that the increased CTB of *osmkkk62/70* and *osmkkk55/62/70* mutants might be due to more healthy anthers and the higher fertile pollen ratio under LT treatment.

### 2.4. OsMKKK70 and OsMKKK62 Negatively Regulate GA Contents in Anthers

**Figure 4:**
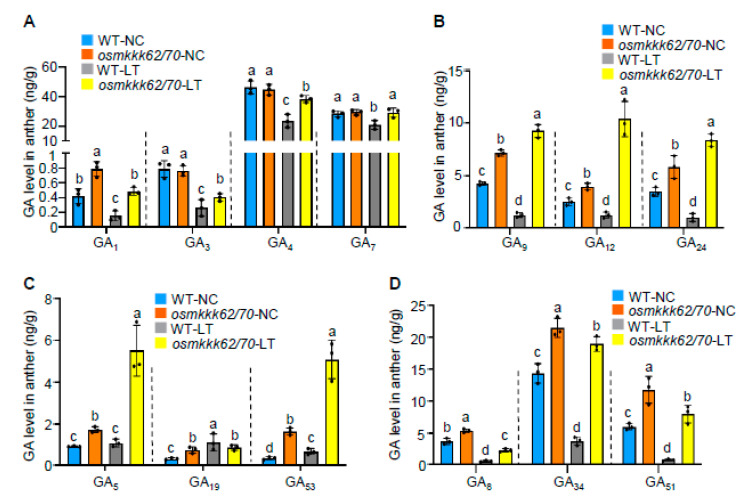
*osmkkk62/70* has higher GA content than WT in anthers under low-temperature treatment. (**A**) Measurements of endogenous bioactive GAs in developing anthers of WT and *osmkkk62/70* plants grown under NC and after LT treatment. Values are means ± SE (n = 3). (**B**–**D**) The endogenous contents of GA biosynthetic precursors (**B**,**C**) and deactivated GAs (**D**) of WT and *osmkkk62/70* plants grown under NC or after LT treatment. Values are means ± SE (n = 3). Each dot represents the result from one biological replicate; error bars indicate means ± SE. Statistically significant differences are indicated by different lowercase letters (*p* < 0.05, one-way ANOVA with Tukey’s significant difference test).

Low-temperature treatment resulted in anther GA contents being markedly decreased and the application of exogenous GA can successfully rescue cold-injured pollen sterility [9,20]. Recently, it was reported that the *oswrky53* mutant displays increased CTB by fine-tuning GA levels in anthers [9]. Moreover, it was also reported that OsMKKK70 functions upstream of OsWRKY53 via activating the OsMAPKK4-OsMAPK6-OsWRKY53 cascade in regulating rice grain size and leaf angle [6]. These results have motivated us to test whether OsMKKK70 also regulates rice CTB by fine-tuning GA contents in anthers. To this end, we quantified the endogenous GA levels in anthers of WT and mutant plants grown under both NC and LT. Given that *osmkkk62/70* and *osmkkk55/62/70* have a similar CTB phenotype, we only measured the GA level of *osmkkk62/70* (Figure 4). GA_1_, GA_3_, GA_4_, and GA_7_ are bioactive GAs, and GA_4_ and GA_7_ are the predominant GAs in reproductive organs, while GA_1_ and GA_3_ are the main GAs in vegetative organs [6]. We found that the *osmkkk62/70* mutant and WT accumulated similar levels of GA_4_ and GA_7_ under NC (Figure 4A). Importantly, following 15 d at 18 °C LT treatment, the levels of GA_4_ and GA_7_ significantly decreased in WT relative to NC, whereas they obviously did not decrease in *osmkkk62/70* (Figure 4A). GA_1_ and GA_3_ contents were slightly decreased after LT treatment in both WT and *osmkkk62/70*, but the *osmkkk62/70* mutant still had a higher level than WT under LT conditions (Figure 4A). The levels of immediate precursors bioactive GA, including GA_9_, GA_12_, GA_24_, GA_5_, GA_19_, and GA_53_, were also higher in the *osmkkk62/70* mutant than those in WT under both NC and LT (Figure 4B,C). Additionally, the *osmkkk62/70* mutant contained significantly more deactivated GAs (GA_8_, GA_34_, and GA_51_) than in WT (Figure 4D). Considering that GA_4_ and GA_7_ are predominant bioactive GAs in reproductive organs, and the *osmkkk62/70* mutant had a higher level of GA_4_ and GA_7_ than in WT after LT treatment (Figure 4A), these results demonstrate that the *osmkkk62/70* mutant might accumulate more bioactive GAs than WT under LT treatment.

**Figure 5:**
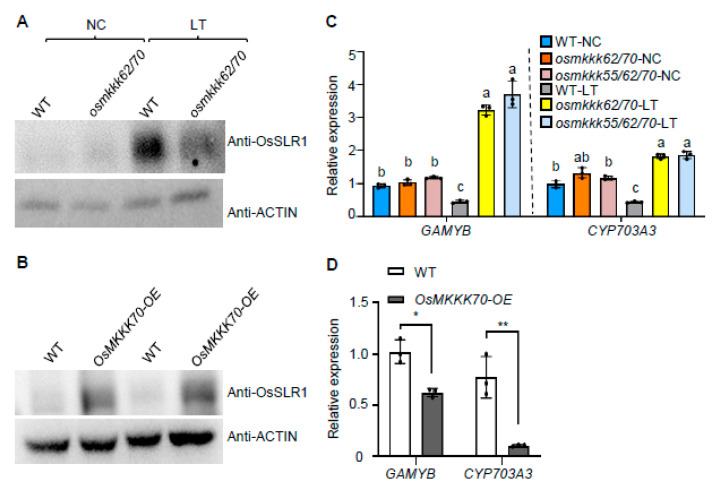
OsMKKK70 negatively regulates CTB by modulating the GA content in anthers. (**A**) OsSLR1 protein level in anthers of WT and *osmkkk62/70* plants grown under NC and after LT treatment for 4 days detected by anti-OsSLR1 antibody. ACTIN contents detected with anti-ACTIN antibody were used as loading control. (**B**) OsSLR1 protein level in anthers of WT and *OsMKKK70-OE* plants grown under NC detected by anti-OsSLR1 antibody. ACTIN contents detected with anti-ACTIN antibody were used as loading control. (**C**) The expression of *GAMYB* and *CYP703A3* in anthers of WT, *osmkkk62/70*, and *osmkkk55/62/70* plants grown under NC and after LT treatment for 4 days. Each dot represents the result from one biological replicate; error bars indicate means ± SE (n = 3). Statistically significant differences are indicated by different lowercase letters (*p* < 0.05, one-way ANOVA with Tukey’s significant difference test). (**D**) The expression of *GAMYB* and *CYP703A3* in anthers of WT and *OsMKKK70-OE* plants grown under NC. Each dot represents the result from one biological replicate; error bars indicate means ± SE (n = 3). *p* values were calculated by Student’s *t*-test. ** *p* < 0.01; * *p* < 0.05.

To confirm this result, we examined the protein level of OsSLR1 (Slender rice 1, rice DELLA protein) in anthers, as OsSLR1 degradation is promoted by GA and the OsSLR1 level can be used as a marker of endogenous GA content [28]. Results showed that the *osmkkk62/70* mutant and WT accumulated similar OsSLR1 protein levels under NC; however, after the LT treatment, the OsSLR1 protein level in the *osmkkk62/70* mutant was markedly lower than that in WT (Figure 5A). Simultaneously, we also examined the OsSLR1 protein level in *OsMKKK70-OE* anthers under NC. The result showed that the OsSLR1 protein level in *OsMKKK70-OE* was higher than that in WT (Figure 5B). *GAMYB* (GA-regulated MYB domain transcription factor gene) and its target gene *CYP703A3* are GA-induced anther specifically expressed genes [29]. We also examined the expression level of these two marker genes and found that the expression levels of *GAMYB* and *CYP703A3* in WT were comparable to those in *osmkkk62/70* and *osmkkk55/62/70* mutants under NC. However, at the LT condition, the expressions of *GAMYB* and *CYP703A3* in *osmkkk62/70* and *osmkkk55/62/70* mutants were significantly higher than those in WT (Figure 5C). Consistent with this result, the expressions of *GAMYB* and *CYP703A3* in *OsMKKK70-OE* were significantly lower than those in WT (Figure 5D). Collectively, these findings suggest that OsMKKK70 and its homologs might negatively regulate rice CTB by modulating the GA content in anther.

### 2.5. OsMKKK70 Negatively Regulates GA Biosynthesis Gene Expression in Anther

**Figure 6:**
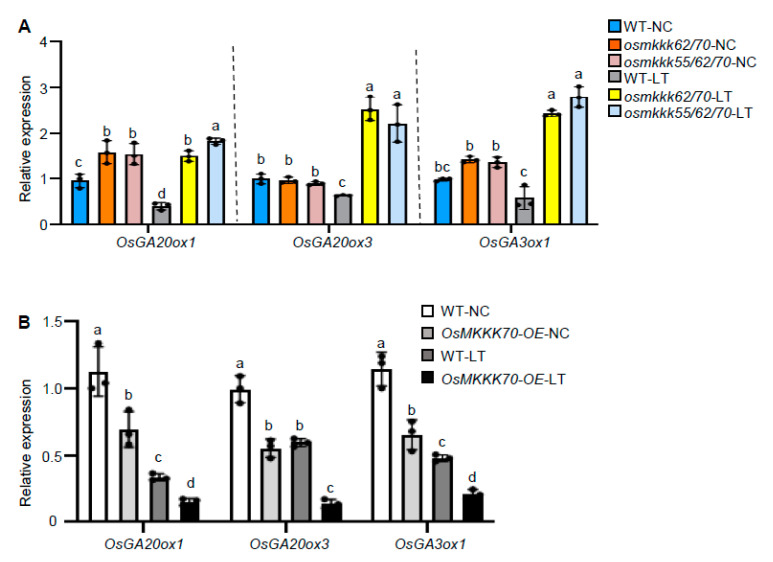
OsMKKK70 negatively regulates GA biosynthesis gene expression in anthers. (**A**) The expression of GA biosynthesis genes in anthers of WT, *osmkkk62/70*, and *osmkkk55/62/70* plants grown under NC and after LT treatment for 4 days. Data are shown as means ± SE (n = 3). (**B**) The expression of GA biosynthesis genes in anthers of WT and *OsMKKK70-OE* plants grown under NC and after LT treatment for 4 days. Data are shown as means ± SE (n = 3). Each dot represents the result from one biological replicate; error bars indicate means ± SE. Statistically significant differences are indicated by different lowercase letters (*p* < 0.05; one-way ANOVA with Tukey’s significant difference test).

In rice, *OsGA20ox1*, *OsGA20ox3*, and *OsGA3ox1* are vital genes that catalyze to generate bioactive GAs and are highly expressed in reproductive organs [16,30,31,32]. It was reported that LT markedly decreased the expression of *OsGA20ox1*, *OsGA20ox3*, and *OsGA3ox1*, and resulted in lower GA content [9]. To address whether OsMKKK70 functions in CTB via regulating GA biosynthesis genes expression, we first checked the expression of these GA biosynthesis genes in WT, *osmkkk62/70*, and *osmkkk55/62/70* mutants. We found that, after LT treatment, the transcript levels of *OsGA20ox1*, *OsGA20ox3*, and *OsGA3ox1* were significantly higher in *osmkkk62/70* and *osmkkk55/62/70* mutants than those in WT (Figure 6A). Given the higher content of GA_12_ in the *osmkkk62/70* mutant relative to WT under both NC and LT (Figure 4B), we also examined the expression level of ent-kaurenoic acid oxidase (*KAO*), which plays key roles in the early steps of GA biosynthesis [16]. The result showed that *osmkkk62/70* and *osmkkk55/62/70* mutants had a higher expression level of *KAO* relative to WT under NC and LT treatment, which partially explained the greater amount of GA_12_ in the *osmkkk62/70* mutant relative to WT (Figure S4). Deactivation of GA is mostly catalyzed by GA 2-oxidase [31]. Given that the *osmkkk62/70* mutant accumulated more deactivated GAs (GA_8_, GA_34_, and GA_51_) than WT (Figure 4D), we also examined the expression of *OsGA2ox* genes. The results showed that *osmkkk62/70* and *osmkkk55/62/70* mutants had higher expression levels of *OsGA2ox1* and *OsGA2ox3* compared to WT under the LT condition (Figure S4). To confirm that OsMKKK70 negatively regulates GA biosynthesis gene expression in anthers, we further examined the GA biosynthesis genes expression in *OsMKKK70-OE* under NC and LT treatment, and found that the transcript levels of *OsGA20ox1*, *OsGA20ox3*, and *OsGA3ox1* in *OsMKKK70-OE* were lower than those in WT under both NC and LT treatment (Figure 6B). Collectively, these results suggest that OsMKKK70 negatively regulates the GA content during cold stress at the booting stage, and consequently regulates the rice CTB.

### 2.6. OsMKKK70 Might Act Upstream of OsWRKY53 in Regulating Rice CTB

**Figure 7:**
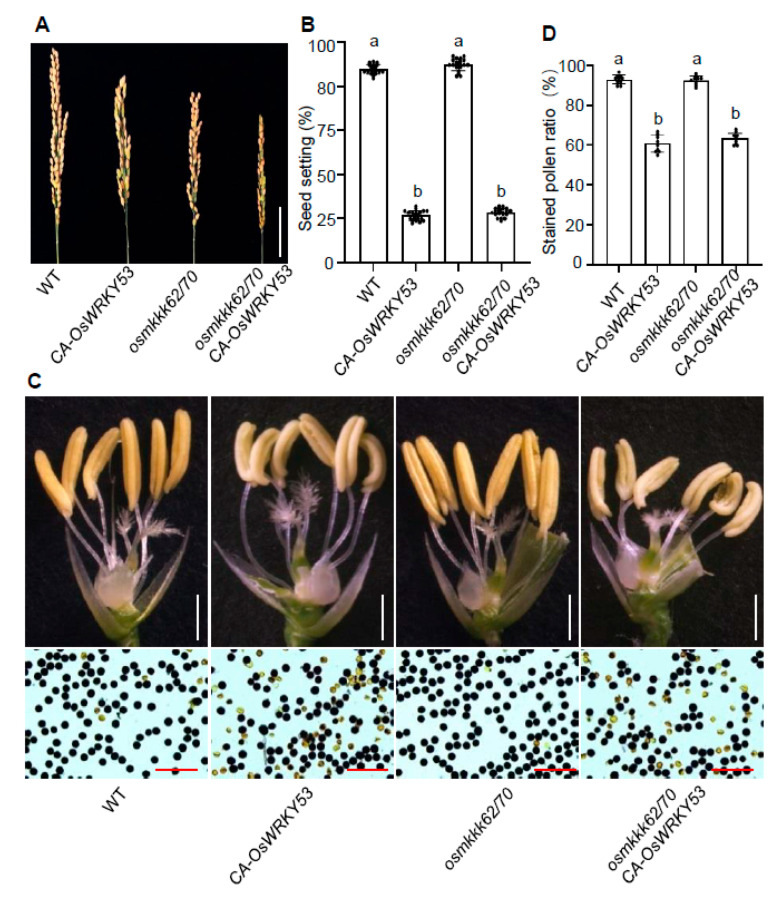
The phenotypic analysis of *osmkkk62/70 CA-OsWRKY53*. (**A**,**B**) Representative images of panicles (**A**) and seed setting rate (**B**) of WT, *CA-OsWRKY53*, *osmkkk62/70*, and *osmkkk62/70 CA-OsWRKY53* plants grown under NC. The scale bar in (**A**) is 5 cm. Data are shown as means ± SE (n = 30). (**C**) Representative images of anthers and stained pollen from WT, *CA-OsWRKY53*, *osmkkk62/70*, and *osmkkk62/70 CA-OsWRKY53* plants grown under NC. The scale bars in the upper and lower images are 1 cm and 50 μm, respectively. (**D**) Quantification of stained pollen ratio in WT, *CA-OsWRKY53*, *osmkkk62/70*, and *osmkkk62/70 CA-OsWRKY53* plants. Data are shown as means ± SE (n = 10). Each dot represents the result from one biological replicate; error bars indicate means ± SE. Statistically significant differences are indicated by different lowercase letters (*p* < 0.05; one-way ANOVA with Tukey’s significant difference test).

MAPK cascades have been shown to function upstream of several group Ia members of WRKY proteins. For example, OsWRKY53 is a phosphorylated substrate of OsMAPK6, and functions downstream of the OsMKKK10-OsMKK4-OsMAPK6 and OsMKKK70-OsMKK4-OsMAPK6 cascades to regulate seed size [6,33,34]. Recently, it has been found that OsWRKY53 negatively regulates rice CTB by fine-tuning GA levels in anther [9]. In this study, we found that *osmkkk62/70* and *osmkkk55/62/70* mutants showed increased CTB phenotypes, including higher seed setting rates and more GA contents in anthers at the LT condition, which is similar to the *oswrky53* mutant as reported (Figure 2A–C, Figure 4A–D and Figure S3) [9]. In addition, *OsMKKK70-OE* lines showed a decreased seed setting rate, unhealthy anther morphology, and lower expression level of GA biosynthesis genes in anthers under NC, which is also similar to *OsWRKY53-OE* plants (Figure 1B–G and Figure S1) [6,9]. These results imply that the OsMKKK70-OsMKK4-OsMAPK6-OsWRKY53 cascade might also be involved in rice CTB. Notably, we found that *smg1-1* (*OsMKK4* mutant) and *dsg1* (*OsMAPK6* mutant) showed comparable relative seed setting rates to WT at the LT condition (Figure S7A–F). This might be due to the decreased seed setting rates of *smg1-1* and *dsg1* under NC (Figure S7A–F). In addition, we also overexpressed the constitutively active (CA) form of *OsWRKY53* in the *osmkkk62/70* background, and generated *osmkkk62/70 CA-OsWRKY53* plants [6]. We found that, compared with WT and *osmkkk62/70*, *osmkkk62/70 CA-OsWRKY53* plants showed a lower seed setting, decreased fertile pollen ratio, and shriveled and pale anther morphology under NC, which is very similar to *OsWRKY53-OE* plants (Figure 7A–D) [6,9]. We further examined the GA biosynthesis genes expression in *osmkkk62/70 CA-OsWRKY53* under NC. We found that the transcript levels of *OsGA20ox1*, *OsGA20ox3*, and *OsGA3ox1* in *CA-OsWRKY53* and *osmkkk62/70 CA-OsWRKY53* were comparable, which were lower than those in the WT and *osmkkk62/70* (Figure S8). These results indicated that OsMKKK70 might function upstream of OsWRKY53 in regulating rice CTB.

## 3. Discussion

In this study, we demonstrated that OsMKKK70 and its homologs might be novel negative regulators of CTB by fine-tuning GA levels in anther. First, *OsMKKK70* expression is rapidly induced by cold treatment at the booting stage (Figure 1A), and the *OsMKKK70-OE* plant is more sensitive to cold stress at the booting stage (Figure 1B–G and Figure S1). Second, *osmkkk62/70* and *osmkkk55/62/70* mutants have increased CTB due to higher pollen fertility and healthier anther morphology (Figure 2, Figure 3 and Figure S3). Third, the *osmkkk62/70* mutant displays higher GA contents relative to WT at the LT condition (Figure 4 and Figure 5). Fourth, OsMKKK70 negatively regulates GA biosynthesis, and then fine-tunes the GA contents during cold stress at the booting stage (Figure 6). Taken together, these results demonstrate that OsMKKK70 and its homologs might negatively regulate CTB by modulating the GA content in anthers.

### 3.1. OsMKKK70, OsMKKK62, and OsMKKK55 Might Function Redundantly in Regulating Rice CTB

MKKKs function at the top level of MAPK cascades and play indispensable roles in regulating plant growth, development, and stress responses. Rice contains approximately 75 OsMKKK family members [25,26,27]. Our previous study demonstrated that *OsMKKK70* has two homologs (*OsMKKK62* and *OsMKKK55*) with high sequence similarity and these three OsMKKK genes function redundantly to regulate seed size and leaf angle in rice [6]. In this study, we discovered that *osmkkk55*, *osmkkk62*, and *osmkkk70* mutants show no observable phenotype compared with WT in CTB (Figure S2), but *osmkkk62/70* double and *osmkkk55/62/70* triple mutants display increased CTB (Figure 2, Figure 3 and Figure S3). Notably, unlike *OsMKKK70-OE*, *OsMKKK55-OE* and *OsMKKK62-OE* have a comparable seed setting rate to WT, and the expression of *OsMKKK55* and *OsMKKK62* was not obviously induced during the booting stage under the LT condition (Figure S5A–D). In addition, we also discovered that *OsMKKK62* and *OsMKKK55* have higher levels in anther (Figure S6A,B). These results indicated that OsMKKK70 might function redundantly with its homologs in regulating rice CTB, and OsMKKK70 plays the dominant role. However, it cannot be ruled out that OsMKKK70 and two homologs (OsMKKK62 and OsMKKK55) function in different pathways for CTB.

### 3.2. OsMKKK70-OsMKK4-OsMAPK6-OsWRKY53 Cascade Might Mediate a Trade-Off between Grain Size and CTB

Grain size and CTB are critical determinants of final grain yield. In our previous work, we found that overexpressing *OsMKKK70* caused plants to produce larger seeds, the *osmkkk62/70* double mutant and the *osmkkk55/62/70* triple mutant displayed significantly smaller seeds [6]. Similarly, *OsWRKY53* overexpression led to enlarged increased grain size, in contrast to the smaller seeds in the *oswrky53* mutant [34]. We also demonstrated that OsMKKK70 regulates rice grain size through the OsMKK4-OsMAPK6-OsWRKY53 cascade signaling pathway [6]. Moreover, we also reported that the *oswrky53* mutant shows an increased CTB phenotype via tuning the GA level in anthers [9]. In this study, we found that *osmkkk62/70* and *osmkkk55/62/70* mutants show an increased CTB phenotype (Figure 2A–C, Figure 4A–D and Figure S3). In addition, overexpressing *CA-OsWRKY53* can rescue the small seed size of *osmkkk62/70* [6]. This study shows that the seed setting, fertile pollen ratio, anther morphology, and GA biosynthesis genes expression level of the *osmkkk62/70 CA-OsWRKY53* plant are very similar to those of *CA-OsWRKY53* plants under NC (Figure 7A–D and Figure S8). These results indicated that the OsMKKK70-OsMKK4-OsMAPK6-OsWRKY53 cascade might mediate a trade-off between grain size and CTB.

To support OsMKKK70 negatively regulating rice CTB by modulating the GA content in anther, we examined the expression level of GA biosynthesis genes in *OsMKKK70-OE* and WT plants under NC and after LT treatment for 4 days. The result showed that the expression levels of GA biosynthesis genes in *OsMKKK70-OE* are lower than those in WT under NC (Figure 6B). After LT treatment, the expression levels of GA biosynthesis genes in *OsMKKK70-OE* show a greater degree of reduction, which are even lower than those in WT (Figure 6B). Simultaneously, the level of OsSLR1 protein, GA-induced genes, and GA biosynthesis genes is comparable between the *osmkkk70* mutant and WT under NC and after LT treatment, which might result from functional redundance (Figure S9).

Notably, unlike *oswrky53* showing no accompanying yield penalty, *osmkkk62/70* and *osmkkk55/62/70* mutants exhibit a decreased grain yield under NC (Figure S10A,B). The grain number per panicle and 1000-grain weight of the *osmkkk62/70* and *osmkkk55/62/70* mutants are significantly lower than those of WT (Figure S10A,B). In addition, *osmkkk62/70* and *osmkkk55/62/70* mutants show reduced plant height and panicles length compared with WT, which are also limiting factors for direct application (Figure S10A,B). Although the application potential of *osmkkk62/70* and *osmkkk55/62/70* mutants is not as good as the *oswrky53* mutant, the function characterization of OsMKKK70 in CTB provides a novel target for cold tolerance breeding in rice.

## 4. Materials and Methods

### 4.1. Plant Materials and Growth Conditions

Rice (*Oryza sativa* ssp. *japonica*) cultivar Longjing 11 (LJ11) was used to generate the *OsMKKK70* transgenic plants and as the WT control. *OsMAPK6* mutant *dsg1* [35] and *OsMKK4* mutant *smg1-1* [36] were used to perform cold tolerance at booting stage (CTB) assays. Rice cultivars, SF43 and ZH11 (*Oryza sativa* ssp. *japonica*), were used as the wild-type control to compare with the corresponding mutants in CTB assays. The plants were cultivated in an experimental field (Heilongjiang Province, China) under natural long-day conditions or in a growth chamber at 30 °C for 14 h (light) and 24 °C for 10 h (dark).

### 4.2. Generation of Transgenic Rice Plants

The *osmkkk55*, *osmkkk62*, *osmkkk70*, *osmkkk62/70*, and *osmkkk55/62/70* mutants generated by genome editing via clustered regularly interspaced short palindromic repeats (CRISPR)/CRISPR-associated nuclease 9 (Cas9) technology were used for analysis in this study and were described previously [6,37]. Two independent *osmkkk62/70* mutants, *osmkkk62/70-1* and *osmkkk62/70-2*, were identified [6]. In this study, *osmkkk62/70-2* was used for most of the experiments and named as *osmkkk62/70* if not indicated. The overexpression plants of *OsMKKK70* were generated as described previously [6]. In order to generate constitutively active *OsWRKY53* (*CA-OsWRKY53*) and *osmkkk62/70 CA-WRKY53* transgenic plants, the full-length coding sequence of *OsWRKY53* in which the five conserved Ser residues in the SP cluster were mutated to Asp was cloned into *pCAMBIA2300*. The construct was, respectively, transformed into rice cultivar Longjing 11 and *osmkkk62/70* via Agrobacterium-mediated transformation as described previously [6,38].

### 4.3. Cold Tolerance at Booting Stage (CTB) Assays

The CTB assay was performed in a greenhouse as described previously with [9,10,20] with some modifications. We selected germinated seedlings with a similar vigor of wild-type and mutant plants, cultivated them in pots containing a mixture of soil and water with fertilizer, and grew them in the greenhouse for normal growth (30 °C for 14 h (day) and 24 °C for 10 h (night)). For the point of low-temperature treatment, we established an internal reference with the distance between the auricles of the flag leaf and the penultimate leaf of the main panicles. When the distance between the auricles of the flag leaf and the penultimate leaf of the main panicle was within −5~0 cm, the main panicles were labeled and then transferred to a low-temperature greenhouse (15 °C constant temperature, 14 h light and 10 h dark photoperiod). After the indicated number of days of cold treatment, treated plants were returned to the greenhouse for normal growth until maturity, at which point we harvested and measured traits on labeled main panicles. The seed setting rate of labeled panicles were investigated for evaluating cold tolerance. The relative seed setting rate is the percentage of seed setting rate under low-temperature treatment relative to normal conditions. More than 10 plants per line were used for treatment in each biological replicate.

For RT-qPCR analysis of cold-regulated gene expression, and detecting OsSLR1 protein level, NC sampling was taken on the beginning day of low-temperature treatment, and LT sampling was taken on 4 days after low-temperature treatment described as above.

For measuring GA contents in cold-treated anthers, when the distance between the auricles of the flag leaf and the penultimate leaf of the main panicles was −8 ~−6 cm, LT samples were the plants transferred to the low-temperature greenhouse (18 °C, 14 h light and 10 h dark photoperiod) for continuous growing until around 15 d. NC samples were the plants growing under the normal condition until 1 d before flowering (around 11 d).

### 4.4. Microscopy

Starch staining of pollen grains and observations of anther morphology were performed 1 d before flowering. Anther morphology was observed directly under a microscope (Olympus, Shanghai, China, SZX16). For measuring pollen fertility, anthers were fixed in a formalin-acetic acid-alcohol (FAA) solution. Fixed anthers were ground to release pollen grains, stained with 1% I_2_-KI solution, then observed under a light microscope (Olympus, Shanghai, China, BX53). Blue-stained pollen grains were counted to determine pollen fertility.

### 4.5. Quantification of GAs

For measuring GA contents in cold-treated anthers, when the distance between the auricles of the flag leaf and the penultimate leaf of the main panicles was −8~−6 cm, LT samples were the plants transferred to the low-temperature greenhouse (18 °C, 14 h light and 10 h dark photoperiod) for continuous growing until around 15 d. NC samples were the plants growing under the normal condition until 1 d before flowering (around 11 d). The anthers of *osmkkk62/70* and wild-type plants were harvested 1 d before flowering and stored at −80 °C until further use. The contents of endogenous GAs in anthers were measured by Wuhan Greensword Creation Technology Company (Wuhan, China) (http://www.greenswordcreation.com), referring to the method previously described [39]. Each series of experiments was performed in biological triplicates.

### 4.6. Total RNA Isolation and RT-qPCR Analysis

Total RNA was extracted from the samples using Trizol (Invitrogen, Carlsbad, CA, USA). Reverse transcription was carried out with SuperscriptII Reverse Transcriptase (Invitrogen, USA) using 1000 ng of total RNA. Real-time PCR was conducted with a Lightcycler 480 using SYBR Green PCR master mix (Takara, Kyoto, Japan). The rice ubiquitin gene was used as an internal reference to normalize all gene expression data. Three biological repeats were performed for each analysis. Values are means ± SE of three biological repeats. The primers used are listed in Table S1.

### 4.7. Protein Gel Blot Analysis

For analysis of the protein level of OsSLR1, the total proteins were extracted using the protein extraction buffer (25 mM Tris–HCl (pH 7.0), 10 mM MgCl2, 10 mM NaCl, and 5 mM DTT). The protein samples were separated by 8% SDS-PAGE and detected by immunoblot analysis with anti-OsSLR1 antibody [40] and anti-ACTIN antibody (Abmart, Shanghai, China; M20009M).

### 4.8. Statistical Analysis

Statistically significant differences were determined by Student’s *t*-test and one-way ANOVA. The means and SE were based on independent biological samples.

### 4.9. Accession Numbers

Sequence data from this article can be found in the Rice Genome Annotation Project under the following accession numbers: *OsMKKK70* (LOC_Os01g50410); *OsMKKK62* (LOC_Os01g50420); *OsMKKK55* (LOC_Os01g50400); *OsMKK4* (LOC_Os02g54600); *OsWRKY53* (LOC_Os05g27730); *OsMAPK6* (LOC_Os06g06090); *OsSLR1* (LOC_Os03g49990); *GAMYB* (LOC_Os01g59660); *CYP703A3* (LOC_Os08g03682); *KAO* (LOC_Os06g02019); *OsGA20ox-1* (LOC_Os03g63970); *OsGA20ox-3* (LOC_Os07g07420); *OsGA3ox-1* (LOC_Os05g08540); *OsGA2ox-1* (LOC_Os05g06670); *OsGA2ox-3* (LOC_Os01g55240); *Ubiquitin* (LOC_Os01g22490).

## Data Availability

All data supporting the findings of this study are available from the corresponding author on reasonable request.

## Supplementary Materials

The following supporting information can be downloaded at: https://www.mdpi.com/article/10.3390/ijms232214472/s1.

## Abbreviations

CTB	cold tolerance at booting stage
GA	gibberellic acid
LT	low temperature
NC	normal condition
ROS	reactive oxygen species
ABA	abscisic acid
