# Supplementary material for: OsMKKK70 Negatively Regulates Cold Tolerance at Booting Stage in Rice
Source: Int J Mol Sci. 2022 Nov 21;23(22):14472. doi: 10.3390/ijms232214472 (PMC9697274; doi:10.3390/ijms232214472)
Supplement: Supplementary file 1 [file ijms-23-14472-s001.zip › ijms-2010402-supplementary/Supplementary Figures.pdf]

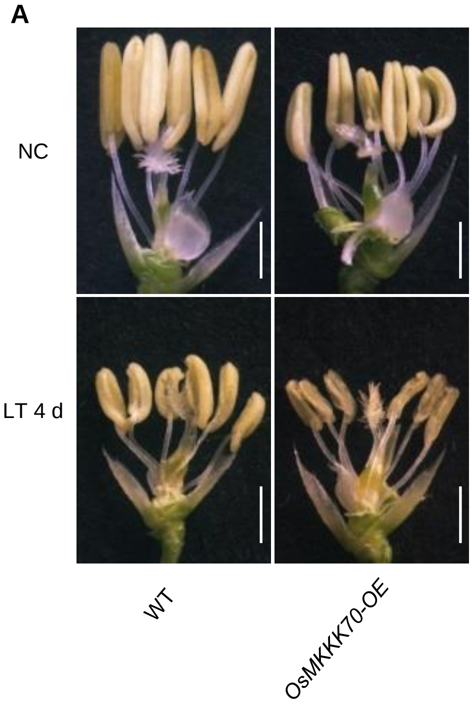

Figure S1. Gross morphology of anthers from WT and *OsMKKK70-OE* plants.
(A) Gross morphology of anthers from WT and *OsMKKK70-OE* plants grown under NC, and after LT treatment for 4 days. The scale bars are 1 cm.

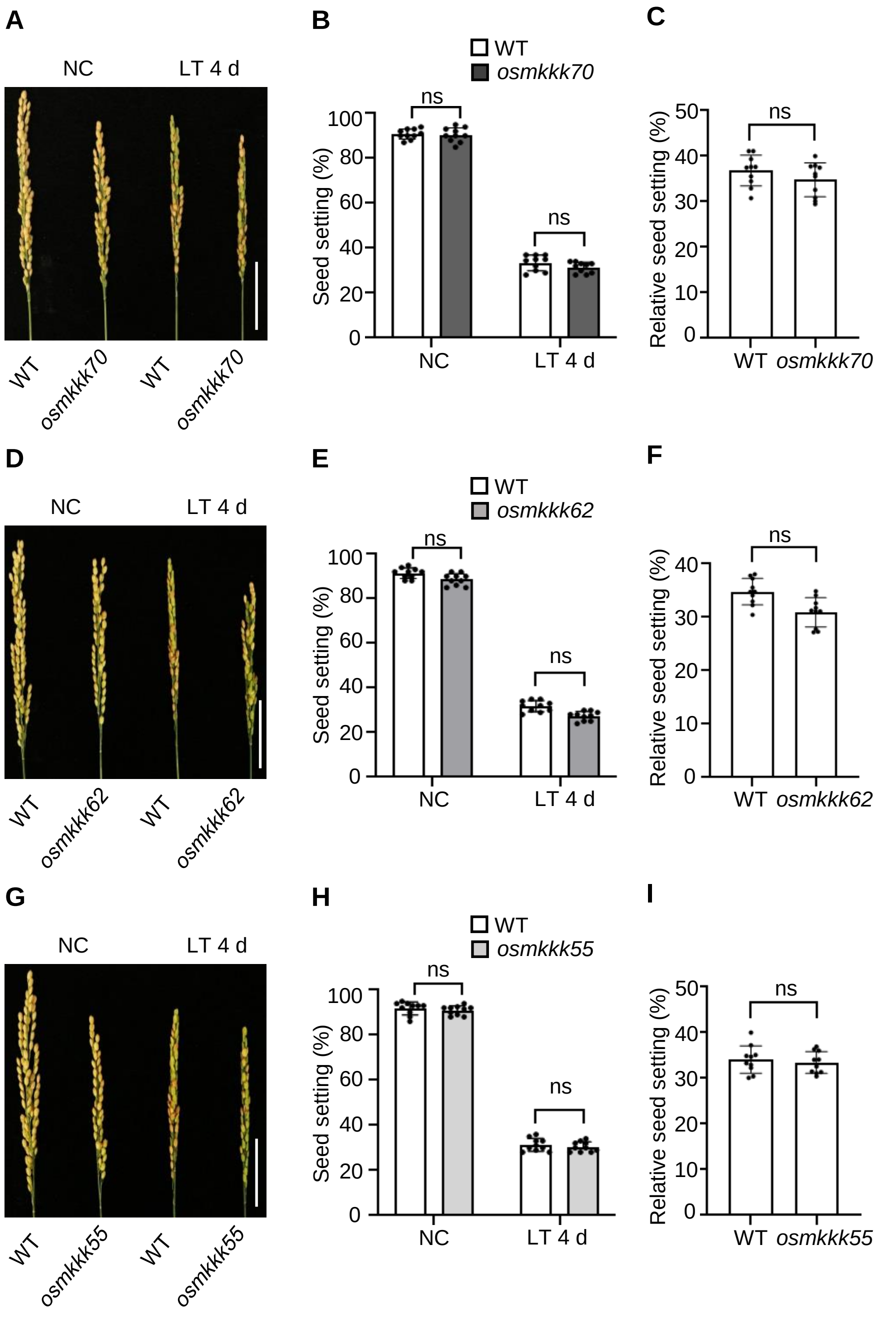

Figure S2. *osmkkk55*, *osmkkk62* and *osmkkk70* mutants cannot improve cold tolerance at the booting.

(A, B, C) Representative images of panicles (A), seed setting rate (B) and relative seed setting rate (C) of WT and *osmkkk70* mutants grown under NC, and after LT treatment for 4 days. The scale bar in (A) is 5 cm. Data are shown as means ± SE (n=10).

(D, E, F) Representative images of panicles (D), seed setting rate (E) and relative seed setting rate (F) of WT and *osmkkk62* mutants grown under NC, and after LT treatment for 4 days. The scale bar in (D) is 5 cm. Data are shown as means ± SE (n=10).
(G, H, I) Representative images of panicles (G), seed setting rate (H) and relative seed setting rate (I) of WT and *osmkkk55* mutants grown under NC, and after LT treatment for 4 days. The scale bar in (I) is 5 cm. Data are shown as means ± SE (n=10).
Each dot represents the result from one biological replicate, error bars indicate means ± SE. P values were calculated by Student's *t*-test. ns indicates no significant difference.

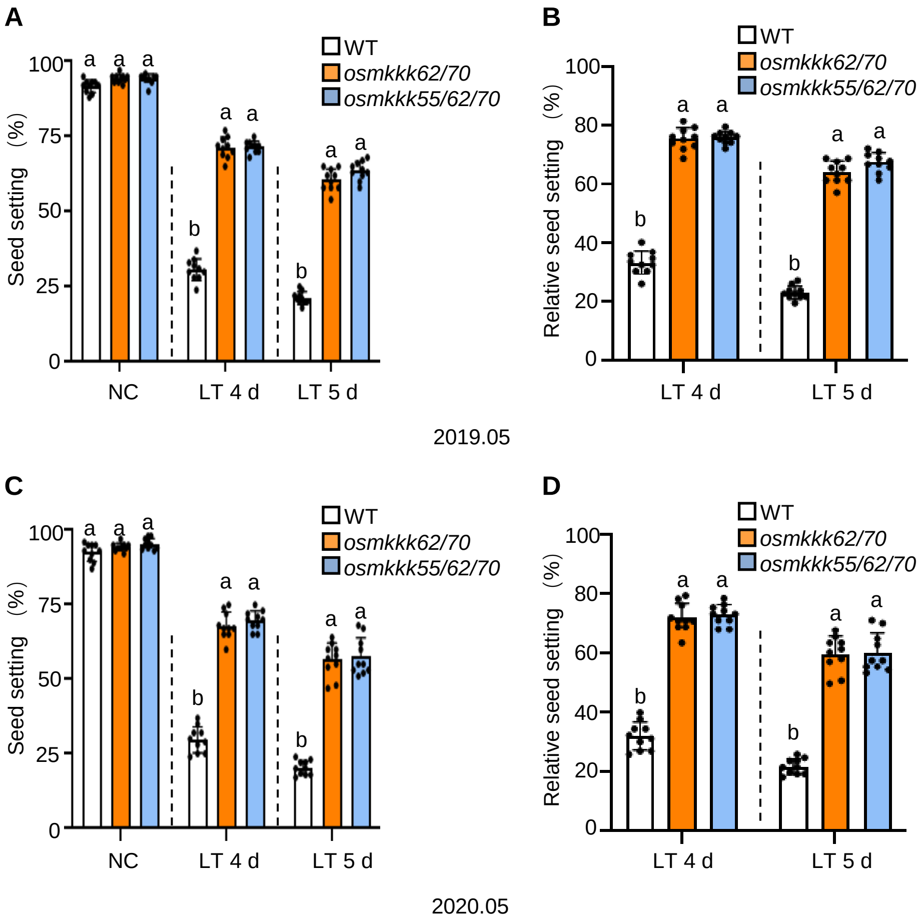

Figure S3. *osmkkk62/70* and *osmkkk55/62/70* shows enhanced cold tolerance at the booting stage in multiple independent replications.

(A-D) The seed setting rate, (A, C) and relative seed setting ratio (B, D) of WT, *osmkkk62/70* and *osmkkk55/62/70* plants grown under NC and various cold stress treatments (LT) are shown separately. The date of each independent cold stress experiment is indicated. Data are shown as means ± SE (n=10).

Dotted lines were drawn to separate the NC and LT, and the comparison is among different genotypes in the same condition. Each dot represents the result from one biological replicate, error bars indicate means ± SE. Statistically significant differences are indicated by different lowercase letters ($P < 0.05$, one-way ANOVA with Tukey's significant difference test).

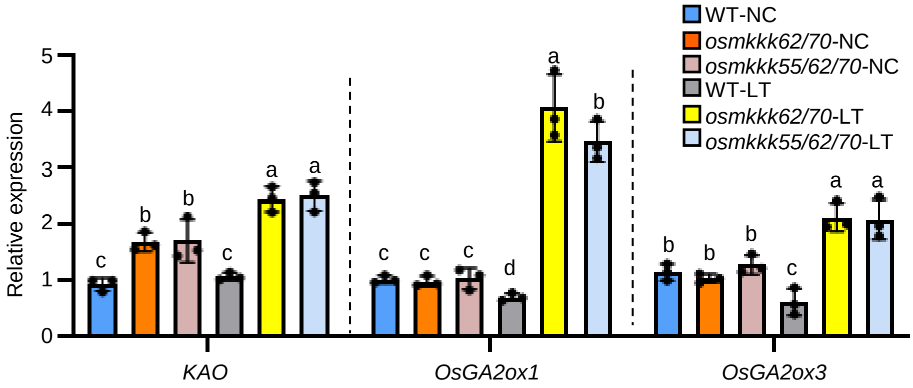

Figure S4. The expression of GA related genes in developing panicles of WT, *osmkkk62/70* and *osmkkk55/62/70* plants grown in NC and exposed to 4 d of LT treatment. The expression levels of GA biosynthesis gene *KAO*, GA catabolism genes *OsGA2ox1* and *OsGA2ox3* in anthers of WT, *osmkkk62/70* and *osmkkk55/62/70* plants grown in normal conditions (NC) and exposed to 4 d of 15° C cold treatment (LT). Data are shown as means ± SE (n=3).

Each dot represents the result from one biological replicate, error bars indicate means±SE. Statistically significant differences are indicated by different lowercase letters ($P < 0.05$, one-way ANOVA with Tukey's significant difference test).

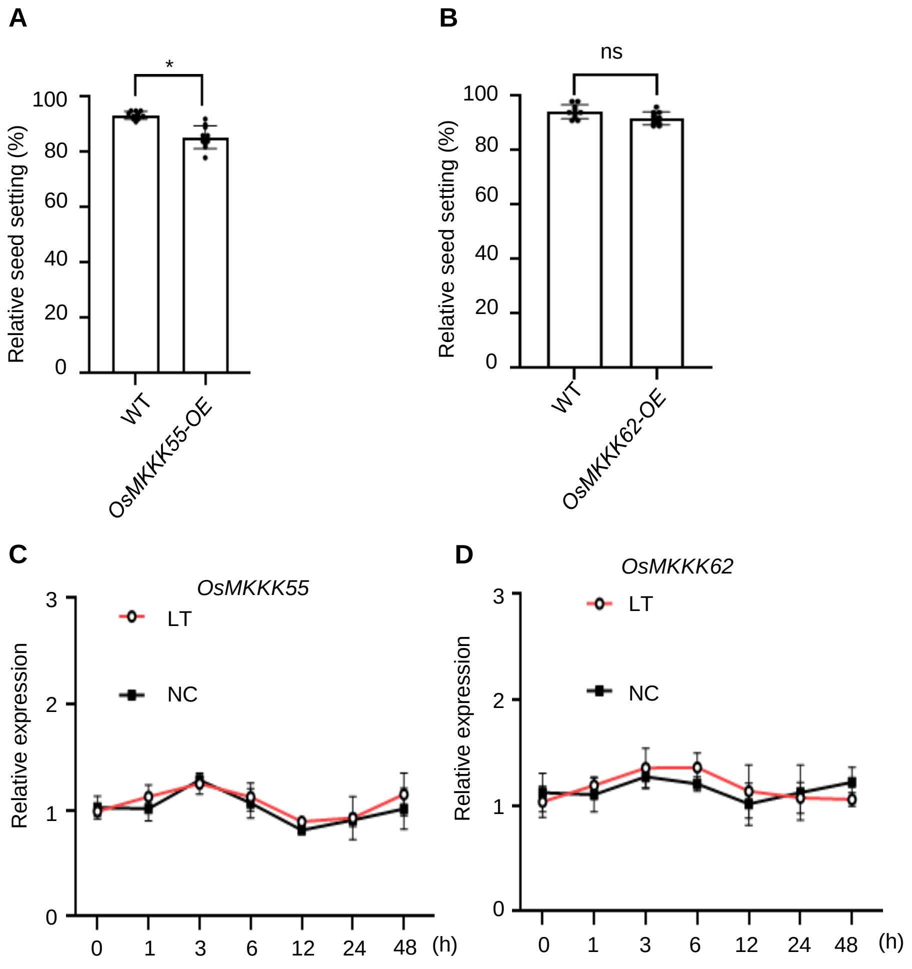

Figure S5. The seed setting rate of *OsMKKK55-OE* and *OsMKKK62-OE* and expression levels of *OsMKKK55* and *OsMKKK62*.

(A) and (B) seed setting rate of *OsMKKK55-OE* and *OsMKKK62-OE* plants grown under NC. Data are shown as means ± SE (n=10).

(C) and (D) Time course of *OsMKKK55* and *OsMKKK62* relative expression in panicles of WT exposed to a 15°C cold treatment during the booting stage. The expression level on 0 hour was set to 1. Data are shown as means ± SE (n=3).

Each dot represents the result from one biological replicate, error bars indicate means ± SE. P values were calculated by Student's *t*-test. * is P<0.05. ns indicates no significant difference.

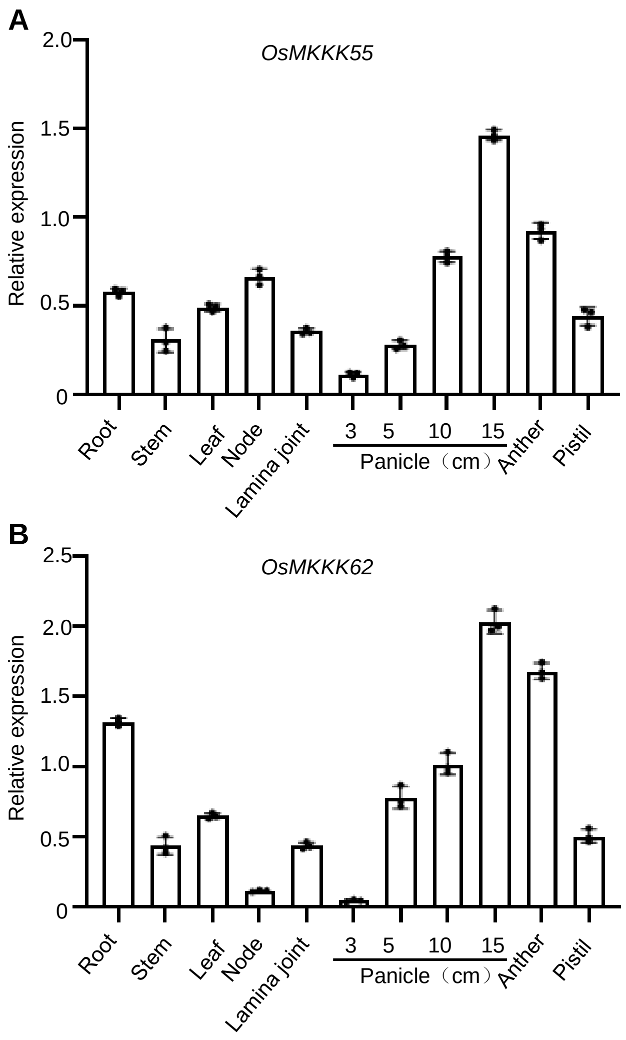

Figure S6. The tissue expression pattern of *OsMKKK55 and OsMKKK62*.

(A) and (B) RT-qPCR analysis of *OsMKKK55* and *OsMKKK62* expression level in different tissues. Each dot represents the result from one biological replicate, error bars indicate means ± SE.

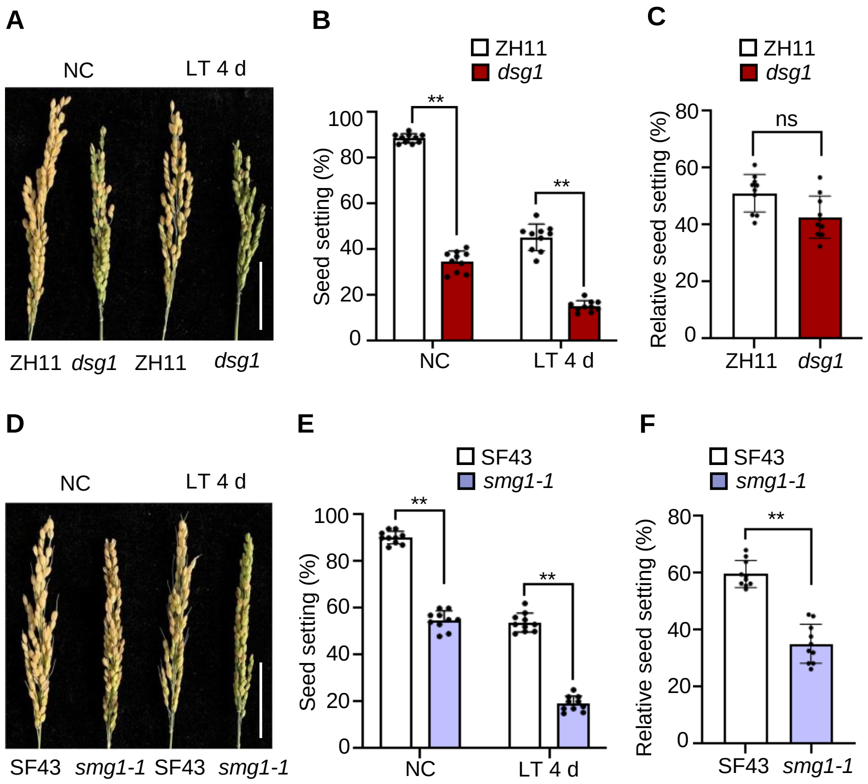

Figure S7. The cold tolerance phenotypes of *dsg1* and *smg1-1* mutants and relative wild type plants.

(A, B, C) Representative images of panicles (A), seed setting rate (B) and relative seed setting rate (C) of ZH11 and *dsg1* mutants grown under NC, and after 4 d LT treatments. The scale bar in (A) is 5 cm. Data are shown as means ± SE (n=10).

(D, E, F) Representative images of panicles (D), seed setting rate (E) and relative seed setting rate (F) of SF43 and *smg1-1* mutants grown under NC, and after 4 d LT treatments. The scale bar in (D) is 5 cm. Data are shown as means ± SE (n=10).

Each dot represents the result from one biological replicate, error bars indicate means ± SE. P values were calculated by Student's *t*-test, ** is P<0.01. ns indicates no significant difference.

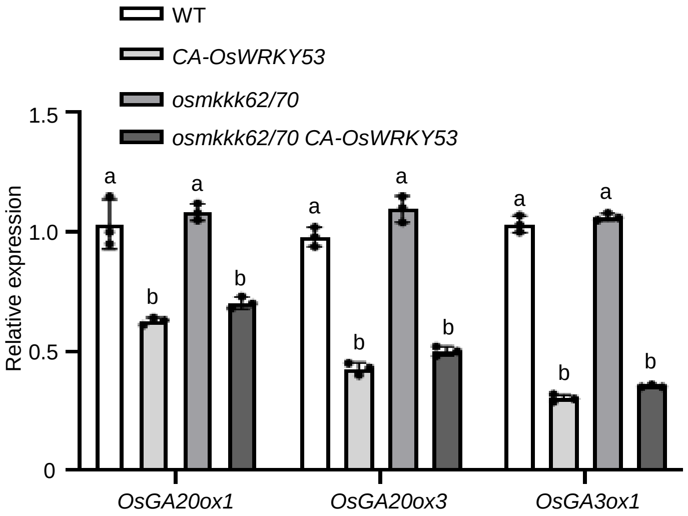

Figure S8. The expression level of GA biosynthesis genes in anthers of *osmkkk62/70 CA-OsWRKY53* plants.

The expression level of GA biosynthesis genes in anthers of WT, *CA-OsWRKY53*, *osmkkk62/70* and *osmkkk62/70 CA-OsWRKY53* plants grown in NC. Data are shown as means ± SE (n=3).

Each dot represents the result from one biological replicate, error bars indicate means ± SE. Statistically significant differences are indicated by different lowercase letters ($P < 0.05$, one-way ANOVA with Tukey's significant difference test).

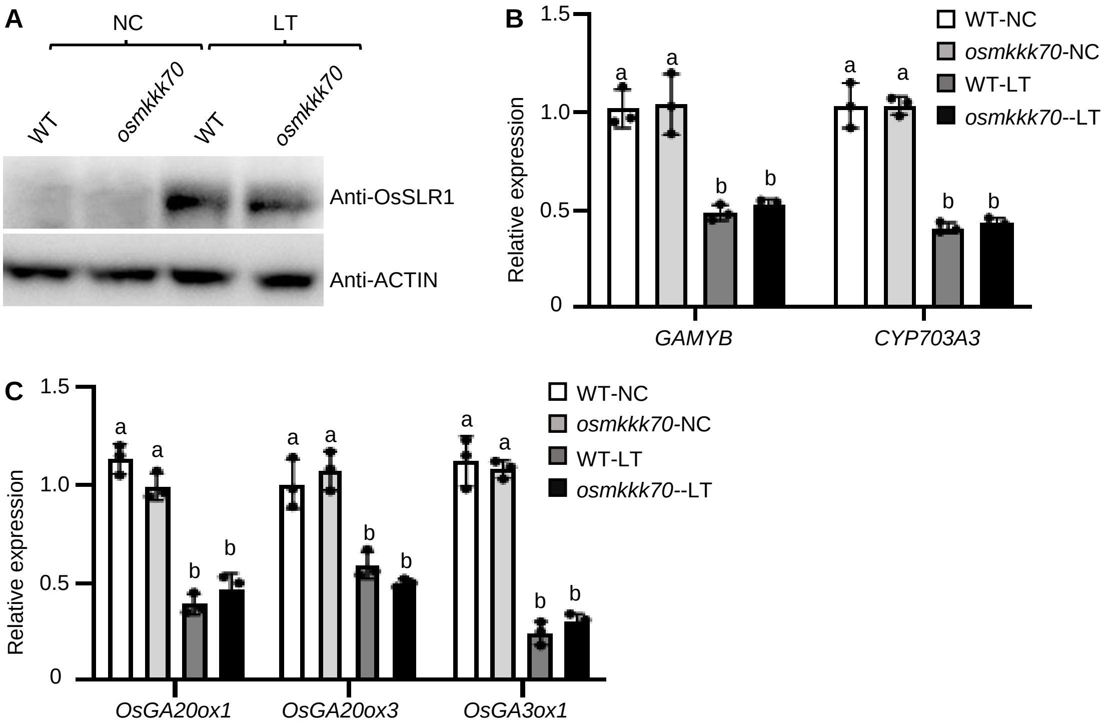

Figure S9. OsSLR1 protein level and GA related genes expression level of *osmkkk70* under NC and after 4 days of LT treatment.

(A) OsSLR1 protein level in anthers of WT and *osmkkk70* plants grown in NC and after LT treatment for 4 days detected by anti-OsSLR1 antibody. ACTIN contents detected with anti-ACTIN antibody were used as loading control.

(B) The expression of *GAMYB* and *CYP703A3* in anthers of WT and *osmkkk70* plants grown in NC and after LT treatment for 4 days. Data are shown as means ± SE (n=3).

(C) The expression of GA biosynthesis genes in anthers of WT and *osmkkk70* plants grown in NC and after LT treatment for 4 days. Data are shown as means ± SE (n=3).

Each dot represents the result from one biological replicate, error bars indicate means ± SE. Statistically significant differences are indicated by different lowercase letters ($P < 0.05$, one-way ANOVA with Tukey's significant difference test).

A

Agronomic traits (2019.10 Heilongjiang)				
Genotype	Thousand-grain weight (g)	Grain number per panicle	Tiller number per plant	Yield per plant (g)
WT	28.3±0.19 (a)	105.6±0.15 (a)	16.7±0.21 (a)	28.9±0.07 (a)
osmkkk62/70	24.3±0.21 (b)	85.8±0.23 (b)	15.9±0.08 (a)	23.5±0.14 (b)
osmkkk55/62/70	23.9±0.15 (b)	82.5±0.08 (b)	16.2±0.13 (a)	23.1±0.12 (b)

B

Agronomic traits (2020.10 Heilongjiang)				
Genotype	Thousand-grain weight (g)	Grain number per panicle	Tiller number per plant	Yield per plant (g)
WT	29.1±0.23 (a)	110.5±0.16 (a)	19.5±0.15 (a)	33.1±0.16 (a)
osmkkk62/70	25.5±0.06 (b)	89.8±0.14 (b)	20.1±0.12 (a)	26.3±0.09 (b)
osmkkk55/62/70	25.2±0.18 (b)	87.5±0.11 (b)	19.4±0.09 (a)	25.5±0.13 (b)

Figure S10. *osmkkk62/70* and *osmkkk55/62/70* show significant yield penalty.
(A-B) Summary of yield-related traits in WT, *osmkkk62/70* and *osmkkk55/62/70* plants. A and B are two independent replications grown in different years as indicated.
Data are shown as means ± SE (n=30). Statistically significant differences are indicated by different lowercase letters ($P < 0.05$, one-way ANOVA with Tukey's significant difference test).
